# Predicting the Adhesive Layer Thickness in Hybrid Joints Involving Pre-Tensioned Bolts

**DOI:** 10.3390/polym16162284

**Published:** 2024-08-12

**Authors:** Frederico Ricca, Francisco J. Galindo-Rosales, Alireza Akhavan-Safar, Lucas F. M. da Silva, Thomas Fkyerat, Koichi Yokozeki, Till Vallée, Tobias Evers

**Affiliations:** 1 Institute of Science and Innovation in Mechanical and Industrial Engineering (INEGI), Rua Dr. Roberto Frias, 4200-465 Porto, Portugallucas@fe.up.pt (L.F.M.d.S.); 2 Transport Phenomena Research Center (CEFT), Chemical Engineering Department, Faculty of Engineering, University of Porto, Rua Dr. Roberto Frias s/n, 4200-465 Porto, Portugal; 3 ALiCE—Associate Laboratory in Chemical Engineering, Faculty of Engineering, University of Porto, Rua Dr. Roberto Frias s/n, 4200-465 Porto, Portugal; 4 Department of Mechanical Engineering, Faculty of Engineering, University of Porto, Rua Dr. Roberto Frias, 4200-465 Porto, Portugal; 5 ENSTA Bretagne, Dupuy de Lôme Research Institute, 2 rue François Verny, 29806 Brest CEDEX 9, France; thomas.fkyerat@ensta-bretagne.org; 6 Steel Structures Research Laboratory, Nippon Steel Corporation, Futtsu-shi 293-8511, Japan; 7 Fraunhofer Institute for Manufacturing Technology and Advanced Materials IFAM, Wiener Str. 12, 28359 Bremen, Germany; tobias.evers@ifam.fraunhofer.de

**Keywords:** adhesive flow, hybrid joints, pre-tensioned bolts, adhesive layer thickness, CFD

## Abstract

While most academic studies focus on the properties of cured joints, this research addresses the manufacturing process of hybrid joints in their uncured state. Hybrid joints that combine adhesive bonding with pre-tensioned bolts exhibit superior mechanical performance compared to exclusively bonded or bolted joints. However, the adhesive flow during manufacturing in hybrid joints often results in a nonuniform adhesive thickness, where obtaining an exact thickness is crucial for accurate load capacity predictions. This paper presents experiments involving three different adhesives, providing precise measurements of the adhesive layer thickness distribution, which served as a reference when evaluating and validating the subsequent numerical predictions. The numerical predictions were performed using computational fluid dynamics (CFD) to model the flow behavior of the adhesives during the bonding process and their interactions with the metal substrates. The CFD predictions of the adhesive layer thickness showed good agreement with the experimental data, with the relative differences between the average experimental and numerical thickness values ranging from 4.07% to 27.1%. The results were most accurate for the adhesive with sand particles, whose particles remained intact, ensuring that the adhesive’s rheology remained unchanged. The results highlight the importance of the rheological behavior of the adhesive in the final distribution of the adhesive layer thickness, thereby expanding the understanding of these joints.

## 1. Introduction

Adhesive bonding, which plays a crucial role in contemporary engineering, is widely utilized across various industries. Its significance lies in several beneficial attributes; among them is achieving a more uniform stress distribution across the bonded area if compared to mechanical fastening. The value of adhesive bonding is particularly evident in joining dissimilar materials with varying mechanical properties [1]. The concept of hybrid joints, which integrates materials or joining methods, further expands the capabilities of adhesive bonding [2]. This approach enables engineers to utilize an adhesive’s flexibility and high load-bearing capacity, along with the strength and ease of assembly associated with mechanical fasteners [3]. Hybrid joints, which integrate mechanical fasteners with adhesive bonding, present a compelling lap joint technology, offering continuous load distribution and decreasing the reliance on mechanical fasteners. In the aerospace, automotive, and transport industries, hybrid joints can reduce the weight and production costs. On the other hand, the presence of mechanical fasteners ensures structural integrity, even in the event of adhesive failure [4], thus acting as a fail-safe mechanism.

A specific hybrid joint configuration results from the combination of an adhesive layer with pre-tensioned bolts [5]. Applying adhesives to pre-tensioned bolted joints effectively compensates for surface irregularities [6,7,8], thereby reducing the frictional resistance of unbonded bolted joints. In addition, the inclusion of an adhesive layer can reduce the dependence of the joint strength on the preload levels [9,10], allowing greater flexibility in preload control during fabrication and facilitating efficient joining with other materials, such as fiber-reinforced plastic (FRP) [11,12]. In addition, using pre-tensioned bolts in conjunction with adhesive bonding provides immediate strength upon tightening, eliminating the need to wait for the adhesive to cure [13,14]. In addition, pre-tensioned bolts act as a safety measure in the event of a fire [15], where adhesives may fail at typical service temperatures, thereby avoiding issues relating to the long-term performance of bonded joints [16]. In summary, hybrid joints not only strengthen bolted joints but also offer viable alternatives to welded joints, particularly in applications using high-strength steels, which often have lower fatigue strength than static strength [17,18,19].

The origins of research into hybrid joints can be traced back to the 1950s in Germany [20,21,22,23], a view supported by recent studies [9,10,20,24,25]. Despite more than five decades of research, our understanding of hybrid joints formed by the combination of pre-tensioned bolts and adhesive bonding remains incomplete. Empirical evidence strongly emphasizes the superior load-bearing capacity of hybrid joints compared to isolated pre-tensioned bolted or adhesive bonded joints. However, despite extensive research, the precise mechanics governing the interaction between these components remain a subject of ongoing debate. Fundamental questions, such as whether this hybrid joint should be classified as a pre-tensioned bolted joint with increased friction or as a bonded joint, remain unanswered, as emphasized by Yokozeki et al. in their review [26,27].

Unlike in “normal” bonded joints, where the thickness is controlled by spacers [28], glass beads [29], or other means [30], the thickness of the adhesive layer cannot be predetermined in pre-tensioned bolt hybrid joints because the adhesive is applied in a liquid state and its final thickness results from the squeeze flow process [31], which depends on several factors, including the joint geometry (including thickness) and the rheological properties of the adhesive, particularly its viscosity [32]. Precise control of this parameter is critical, as several studies have verified the existence of an optimal adhesive layer thickness, specific to each adhesive, that enhances the mechanical performance of the joint [33,34]. Regarding the nature of the substrate, particularly its roughness, recent evidence suggests that “adhesive flow is independent of the surface condition of the substrate” [35].

The presence of fillers in adhesives [32] introduces additional complexity to the aforementioned flow processes. They not only affect the rheological behavior of the adhesive but also influence the final adhesive layer thickness, depending upon their resistance to crushing, their ability to penetrate the substrate, or if they merely behave as spacers [36]. Despite efforts to estimate or measure it, the precise adhesive layer thickness in hybrid joints remains unclear, with authors describing it as “very thin” [23,37]. This uncertainty is critical as it significantly influences stress, strain [37,38,39], and the load capacity [34,40]. It is also a fundamental parameter needed for any finite element analysis (FEA), particularly strength prediction. Consequently, its numerical prediction avoids carrying out experimental tests, which can be costly and time-consuming.

In general, a bonding process works as follows: a viscous adhesive is applied onto the surface of one substrate in its liquid state, and, subsequently, the other substrate is pressed onto the first one [35]. This causes the adhesive to spread in between and wet both surfaces; the corresponding adhesive flow is called a squeeze flow. In a recent paper [41], an overview of the issues related to the numerical simulation of adhesive spreading for liquid to semi-liquid adhesives was provided, along with a discussion of the advantages and limitations of grid-based and meshless methods in guiding method selection depending on the specific case. In scenarios where the behavior of the fluid directly affects the structure, and vice versa, the need for a fluid–structure interaction (FSI) analysis arises to complement computational fluid dynamics (CFD) calculations. FSI involves the interplay between solid structures and a fluid flow, with the former typically modeled using a Eulerian grid and the latter using a Lagrangian grid [42,43]. FSI simulations are particularly challenging when dealing with large forces, deformations, and nonlinear components [44].

Various numerical methods have been developed to address FSI problems, driven by the increasing demand across scientific and engineering disciplines. The Coupled Euler Lagrangian (CEL) method, which directly couples fluid and solid dynamics within a single simulation model, offers better control over the stability and convergence compared to indirect coupling methods [32,45,46]. Another approach is to use smoothed particle hydrodynamics (SPH), which represents fluids as particles and treats fluid motion within rigid boundaries using various techniques [47,48,49]. Despite producing reasonable results, both methods demonstrate potential for improvement, particularly when dealing with squeeze flow processes, where the predicted normal force generated by the viscous adhesive cannot match the experimental values [46].

Given the complexity and computational cost associated with full FSI simulations, researchers have sought simplified models to assist engineers. One such approach is the use of the Reynolds equation [50], derived from lubricated frictional contacts. It offers closed analytical expressions for simple geometries, like the Stefan [51] and Scott [52] equations for Newtonian and power-law fluids, respectively. Numerical implementations thereof closely resemble the results from 3D-CFD simulations [53], offering high accuracy at much lower computational costs, making them perfect candidates for optimization routines for simplified geometries [54]. While simplified methods offer computational advantages, they may have limitations, particularly with respect to substrate flexibility [55]. Recent research has also explored foundation beam models for the bonded joint strength, such as the analytical model introduced by Cabello et al. [56], which effectively handles nonlinearity in thick flexible bond lines and provides predictive capabilities for different adhesives, stress states, and specimen dimensions [57].

This paper uses CFD tools validated with experimental results to predict the thickness of the adhesive layer in hybrid joints formed by pre-tensioned bolts and viscous adhesives. The focus is on understanding the variables that affect the adhesive flow and layer thickness, including factors such as the joint geometry, the adhesive rheological properties, and the presence of fillers.

## 2. Materials and Methods

### 2.1. Experimental Determination of the Adhesive Layer Thickness

**Figure 1:**
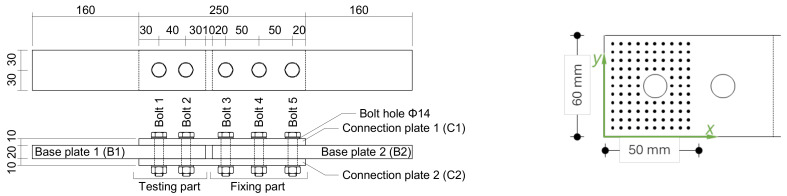
Geometry of the hybrid joints (**left**) and measurement points for the adhesive thickness, distributed uniformly on a 5 mm grid over the connection plates, C1 and C2 (**right**).

All hybrid joints featured corundum blasted S355MC steel plates (as per Figure 1), with inner (base) plates, B1 and B2, which were 20 mm thick, and outer (connection) plates, C1 and C2, which were 10 mm thick; for more details, refer to [5]. Before bolting, all four overlaps were adhesively bonded. The pre-tension of the M12–10.9 high-strength bolts occurred within minutes of adhesive application, squeezing out most of it and leaving only a “very thin” layer. The area of interest in the following is the area with the pair of bolts. This study analyzed three two-part (2K) adhesives selected for steel construction: Scotch-Weld DP490 (DP490), Sikadur 370 (S370), and Scotch-Weld 7240 (SW7240). These adhesives, selected from a larger group of nine, are commonly used in the industry for their superior performance with high-strength steel substrates, making them highly representative of practical applications of hybrid bolted–bonded joints. A dynamic mechanical analysis (DMA) was conducted to measure the glass transition temperature (Tg) of the adhesives. Single lap joint tests on blasted steel substrates were performed to determine the lap shear strength. Tensile tests of the bulk adhesives were carried out to obtain the tensile strength, elastic modulus, and Poisson’s ratio. Additionally, a tensile loading test using a hydraulic jack with a capacity of 1 MN was conducted to determine the joint load capacity. Table 1 summarizes their main properties.

The hybrid joints were prepared by first cleaning the steel surfaces with isopropanol and then blasting them with white fused alumina. Surface roughness measurements were then taken using the MarSurf M300C and PHT 3-350 (Mahr Group, Göttingen, Germany) instruments to provide the Ra and Rz values. The outer 10 mm plate showed Ra values of 7.1 ± 0.3 µm and Rz values of 47.5 ± 4.7 µm, while the inner 20 mm plate showed Ra values of 4.9 ± 0.5 µm and Rz values of 37.1 ± 0.8 µm.

In a departure from conventional methods [6], Marbocote^®^ TRE 45 ECO (Marbocote/Middlewich/England), a release agent used to inhibit adhesion, was brushed onto all interfaces of the inner steel plates to be bonded. The adhesive was then applied evenly to the overlapping areas, aiming for a thickness of approximately 2 mm. Screws with washers were then sequentially inserted and hand-tightened after the wetted joint plates were placed on a table. This process was immediately followed by a full tightening process targeting a torque of 148 N⋅m, equivalent to a force of 64.9 kN in each bolt. When the joints were manually disassembled after full curing for 24 h, the entire adhesive layer remained firmly bonded only to the outer steel sheets, due to the presence of the release agent.

**Figure 2:**
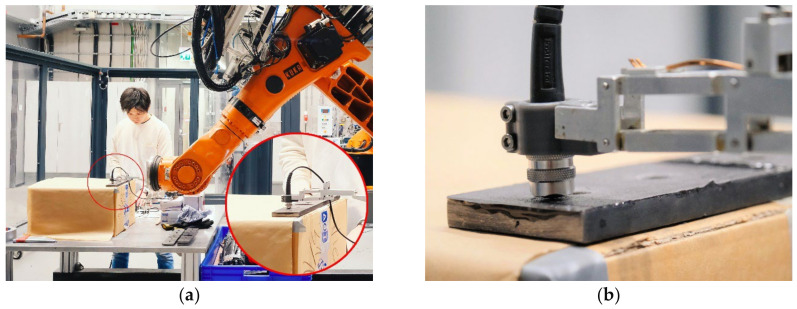
Adhesive layer thickness measurements aided by a robotic handling system programmed for point-to-point movements. (**a**) The robot programmed for thickness measurements; (**b**) a close-up of the thickness measurements.

The thickness of the adhesive layer remaining on the outer fastener plates was then measured using a coating thickness gauge (Positector 6000, DeFelsko, New York, NY, USA). Measurements were taken at grid points on a 5 mm uniform grid, displayed in Figure 2, within a square field around the outer fastener. A robotic handling system was used to ensure accurate contact and positioning, with the gauge mounted on a spring-loaded parallelogram lever attached to the robot flange. The robot (Figure 2), programmed for point-to-point movements, traversed the grid array with three repetitions at each grid point, pausing for 2 s at each point to ensure accurate measurement.

### 2.2. Adhesives and Their Rheological Characterization

As indicated in the Introduction, two adhesive properties are key to determining the thickness of the adhesive layer: the rheological behavior and fillers. The rheology, particularly the viscosity, influences the extrusion dynamics, while fillers can act as spacers and set a minimum thickness.

Viscosity measurements were performed on a TA Instruments DHR-2 rotational rheometer (TA Instruments, New Castle, DE, USA) using parallel plates (Ø25 mm) at 23 °C, the same temperature at which the adhesives were applied in the experimental procedure. The two components of each of the tested adhesives were weighed and mixed according to the manufacturer’s technical datasheet. The freshly mixed adhesive was applied to the lower plate prior to measurement and the upper plate was then lowered for trimming. Measurement gaps of 500 µm were set for DP490 and SW7240. For S370, a gap of 1000 µm was set to reduce the influence of large filler particles on the normal force. The viscosity curve was obtained by imposing a logarithmic shear rate ramp from 0.01 s^–1^ to 100 s^–1^. Data points were evaluated for each shear rate until the adhesive was expelled from the gap, resulting in different maximum shear rates for each adhesive. Fillers were analyzed by thermogravimetric analysis (TGA) using a Discovery TGA Q5000 and microscopy (TA Instruments, New Castle, DE, USA), with the filler content and size detailed in Table 1; further details can be found in [37].

### 2.3. Modeling of the Squeeze Flow

Numerical modeling was conducted using the commercial software Ansys Workbench 2023 R1, featuring the Ansys Fluent 2023 R1 CFD system, a general-purpose computational fluid dynamics (CFD) software program used to model fluid flows, and Ansys Mechanical, a finite element analysis (FEA) software program used to perform structural analysis. The presence of these modules within the Ansys Workbench environment provides a cohesive platform for the coupling of the fluid and solid domains, thereby facilitating the study of FSI phenomena. In the CFD-based models, the adhesive was modeled using the Carreau viscosity model [58], a generalized Newtonian fluid model that expresses the viscosity η as a function of the shear rate $\dot{\gamma }$ through Equation (1). Herein, $\eta_{0}$ is the viscosity at a zero shear rate, $\eta_{\infty }$ is the viscosity at an infinite shear rate, n is a dimensionless power index, and λ is the characteristic time. In the solid model, all relevant mechanical parameters were incorporated. The steel properties included a Young’s modulus of 195,000 MPa and a Poisson’s ratio of 0.33. Plasticity was defined by the yield strength from Table 1, without consideration of work hardening.

(1) $$\eta \left(\dot{\gamma }\right)=\eta_{\infty }+\left(\eta_{0}-\eta_{\infty }\right){\left[1+{\left(\lambda \cdot \dot{\gamma }\right)}^{2}\right]}^{\frac{n-1}{2}}$$

This study considers the isothermal flow of various viscous adhesives when pressed onto metal substrates. Consequently, the energy equation can be omitted from the flow problem, which is governed by the Navier–Stokes equations, i.e., the mass and momentum conservation laws. Considering the adhesive as an incompressible fluid (ρ = constant), the mass conservation equation is simplified as in Equation (2):

(2) ∇ · υ=0,

where υ is the velocity vector. The momentum conservation equation is given by Equation (3):

(3) ρυ ·∇ υ=−∇P+∇·τ,

where *P* is the pressure; the acceleration due to gravity was not considered in the calculations due to its negligible effect on the flow; finally, *τ* stands for the viscous stresses, which are a function of the viscosity and the rate of linear and volumetric deformation that the fluid undergoes.

For squeeze flow simulations, it is necessary to define a moving boundary condition associated with the descending substrate, along with conventional boundary conditions to ensure proper fluid flow. The moving boundary condition was implemented using a user-defined function (UDF) written in the C language (Appendix A). The other conditions used, labeled as conventional conditions, are already available in the software and are listed in Table 2.

Fluent operates based on the finite volume method (FVM), which is optimized for structured meshes as they offer advantages in terms of computational accuracy and numerical efficiency [59,60]. Moreover, the downward movement of the boundary causes deformation in both the fluid domain and the mesh, requiring a dynamic mesh model. For this type of mesh, the dynamic layering method available in Fluent is the most suitable [61], adding or removing layers of cells adjacent to a moving boundary, based on their height. It allows one to specify an ideal layer height, $h_{ideal}$ on each cell adjacent to the moving boundary. While the fluid is being compressed, the program defines the merging of the cells by the height of the cells undergoing compression. If their height reaches a minimum value, $h_{min}$, as a function of the layer collapse factor, $\alpha_{c}$, set by the user, then the cells will merge. Here, $h_{ideal}$ was 80 μm, defined by the height of the mesh elements, and the selected $\alpha_{c}$ was 0.2. When this condition is met, the compressed layer of cells is merged into the layer of cells below the compressed layer.

(4) $$h_{min}<\alpha_{c}\cdot h_{ideal},$$

The FSI simulations were conducted in a transient regime, focusing on assessing substrate deformation and the resulting adhesive layer thickness distribution. This assessment is based on the adhesive’s rheological behavior under squeeze flow conditions, with a predetermined substrate downward velocity. Initially, all adhesives were tested using a downward substrate velocity of 1 mm/s, which was employed in the validation process detailed in Appendix B. The variation in the substrate’s descent speed was employed to match the estimated experimental force at the thinnest adhesive thickness values, as the normal force produced during compression flow is known to be a function of the speed, among other parameters. This adjustment ensured that the simulations accurately captured the behavior of different adhesives across varying thickness ranges while maintaining consistent force application. A time step of 0.01 s was used in all simulations. Table 3 provides an overview of all series run.

The viscosity model chosen is laminar, as the flows under consideration occur at low speeds and involve very high viscosities, resulting in very low Reynolds numbers. The pressure–velocity coupling uses the COUPLED method (Table 4) as it works well in transient simulations and solves the governing equations simultaneously, which leads to a fast convergence rate [62]. The spatial discretization of gradient method chosen was the least squares cell-based method, as it is simple, general, and reliable and it is optimal for a wide range of complex flows [63]. The other spatial and time discretization methods chosen were all of the second order to guarantee the most reliable results, and the stoppage criterion used to end each simulation time step was a residual value of stabilization of 10^−6^.

#### 2.3.1. CFD Models Considered

Two different numerical models were evaluated. The first model validated the selected methodologies (see Appendix B). The second model analyzed the rheological effects of various viscous adhesives on the elastic deformation of the metal substrate and, consequently, on the thickness distribution of the adhesive layer. This model will encompass the CFD and mechanical systems of the problem and will have the geometry of a single bolt hybrid joint.

#### 2.3.2. Fluid–Structure Interaction

The numerical simulation of the fluid–structure interaction (FSI) can be either one-way or two-way. One-way simulations involve information flowing solely from the fluid system to the mechanical system in each iteration, whereas two-way simulations enable bidirectional information exchange, allowing mechanical system changes to influence the simulation results. Two-way simulations are significantly more computationally intensive, especially since all FSI simulations must be conducted in three dimensions, further increasing the computational costs. Given that two-way FSI simulations are primarily necessary when solid deformations significantly impact the fluid flow, a one-way approach was utilized in this study. For information exchange, specific faces where information flows between systems must be selected, corresponding to the adhesive–metal substrate contact areas. In the CFD module, this face is termed the “moving substrate”, while, in the mechanical module, it corresponds to the underside of the joint geometry.

The FSI model comprises a two-step simulation process. In the first step, the simulation addresses the squeeze flow of the adhesive using a geometry identical to that of a hybrid joint featuring a single bolt. In the second step, the simulation involves the interaction between the adhesive flow and the deformation of the metallic substrate.

The geometry of the adhesive and the metal substrate mirrors the most recent experimental work by Yokozeki et al. [37]. Accordingly, the measurements analyzed correspond to a rectangular geometry with a width of 60 mm, a length of 50 mm, an adhesive layer thickness of 3 mm, a steel substrate thickness of 10 mm, and a bolt hole diameter of 14 mm.

**Figure 3:**
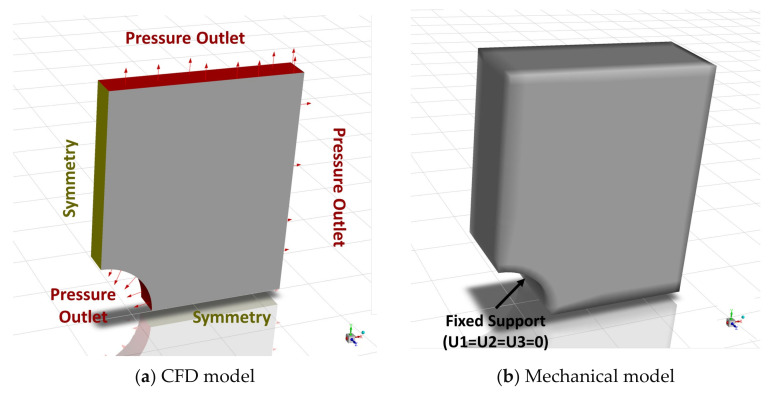
FSI numerical models.

Three-dimensional models are required for FSI simulations. By leveraging the symmetry of the joint geometry, only a quarter of the geometry is represented in the numerical models. In the CFD model, the symmetry conditions are depicted in yellow (Figure 3), while the outlet conditions are shown in red, accompanied by red arrows. Although not visible in Figure 3, the bottom face of the CFD model geometry also has the symmetry condition applied. For the mechanical model, a fixed support condition is applied to the concave face corresponding to the bolt hole.

**Figure 4:**
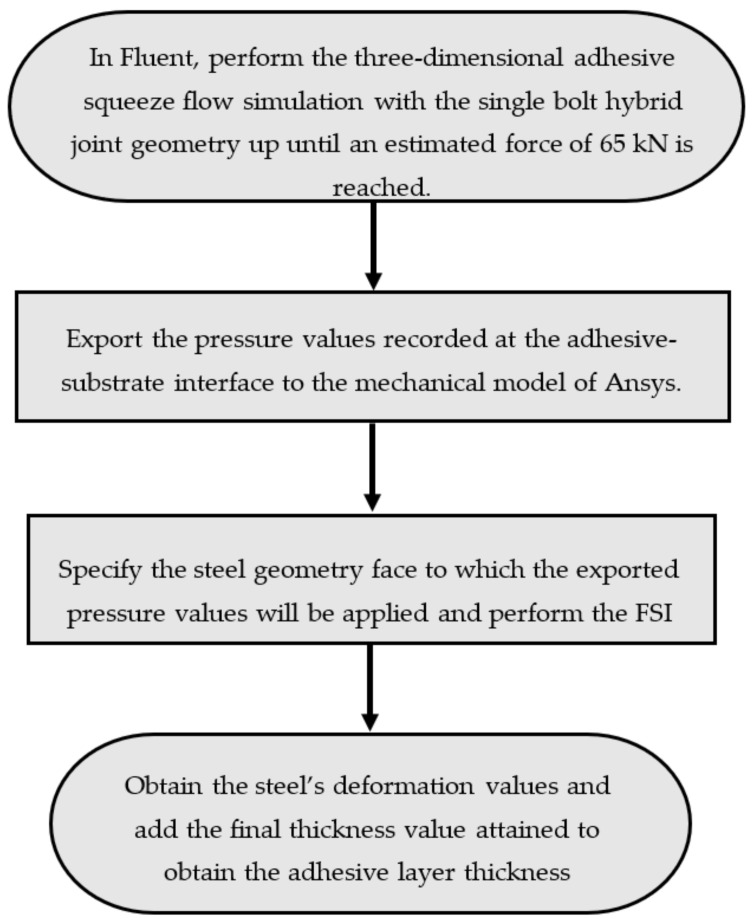
Numerical steps involved in the FSI simulations.

The movement of the substrate is regulated by its speed, *V*. To maintain fidelity to the experimental procedure, the velocity is adjusted to achieve a force of approximately 65 kN at the required thickness or minimum achievable thickness. Implicit control of the force by the speed of the substrate is possible given their relationship in squeeze flows [55]. This ensures a more efficient simulation by ruling out inefficient time periods for adhesive compression associated with the mechanical resistance of the particles and the loosening of the clamping tool. Consequently, the initial thickness of the adhesive is set to values slightly higher than 3 mm for the substrate velocity start-up process. This adjustment ensures that the velocity starts from zero, rather than the final value, to address numerical convergence issues. The steps involved in performing this simulation are summarized in Figure 4.

## 3. Results

### 3.1. Rheological Characterization

**Figure 5:**
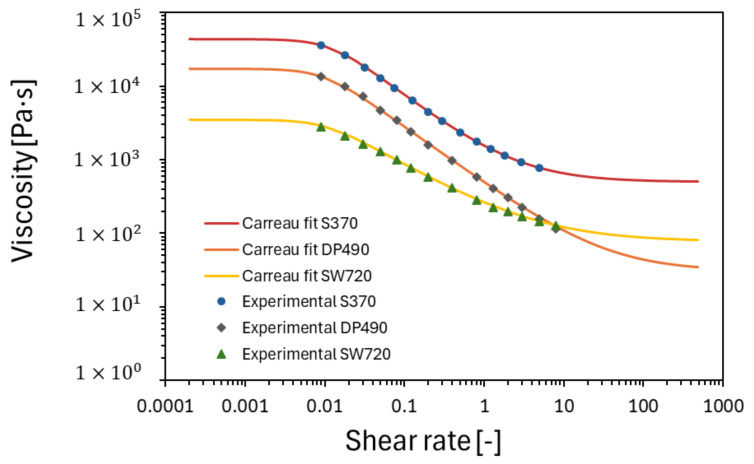
Rheological characterization and Carreau fit.

The steady-state viscosity curves resulting from the evaluation of all tested adhesives are shown in Figure 5. Shear thinning behavior was observed for all adhesives, with higher shear rates leading to a decrease in viscosity. Of the adhesives, S370, which is highly filled, exhibited the highest viscosity at a low shear rate of 0.01 s^–1^. The adhesive SW7240 showed the most pronounced shear thinning effect, with a plateau at very low and high shear rates. For the other three adhesives, the viscosity could be determined at shear rates beyond the plotted data points as the adhesive was displaced from the rheometer gap.

The data resulting from the rheological characterization were then modeled in a Carreau rheological model [58], which expresses the viscosity η as a function of the shear rate $\dot{\gamma }$ through Equation (1). The model parameters were determined by using the Levenberg–Marquardt algorithm [64] in an iterative procedure (Table 5). This algorithm, which combines the Gauss–Newton method and the steepest descent method, works for most cases.

The fitted results provide valuable insights into the rheological behavior of the tested adhesives, each characterized by different viscosity profiles and parameters, starting with the viscosity at a zero shear rate (η_0_), which reflects the viscosity of the adhesives at very low shear rates. For S370, the values are significantly higher compared to DP490 and SW7240, indicating higher flow resistance at low shear rates. This suggests that S370 is more viscous or more resistant to deformation under static conditions compared to DP490 and SW7240. The infinite viscosity ($\eta_{\infty }$), which represents the viscosity of the adhesives at very high shear rates approaching a limit value, is significantly higher for the S370 adhesive when compared to DP490 and SW7240, indicating that they reach higher viscosities under extreme shear conditions. This is due to factors such as the presence of filler particles or the molecular structure of the adhesive.

The time constant (λ) indicates the rate of transition from Newtonian to shear thinning behavior. Higher values of λ indicate a slower transition, meaning that the adhesive maintains its (high) viscosity over a wider range of shear rates before exhibiting shear thinning behavior. Here, S370 has the lowest value, indicating the fastest transition to shear thinning compared to the other adhesives. The power law index (n) characterizes the degree of shear thinning. A lower value of n indicates more pronounced shear thinning. Regarding the adhesives investigated, SW7240 has the strongest shear thinning behavior, followed by DP490 and S370. This suggests that SW7240 is more responsive to changes in the shear rate, resulting in a greater reduction in viscosity with an increasing shear rate compared to the other adhesives.

### 3.2. Adhesive Layer Thickness Measurements

**Figure 6:**
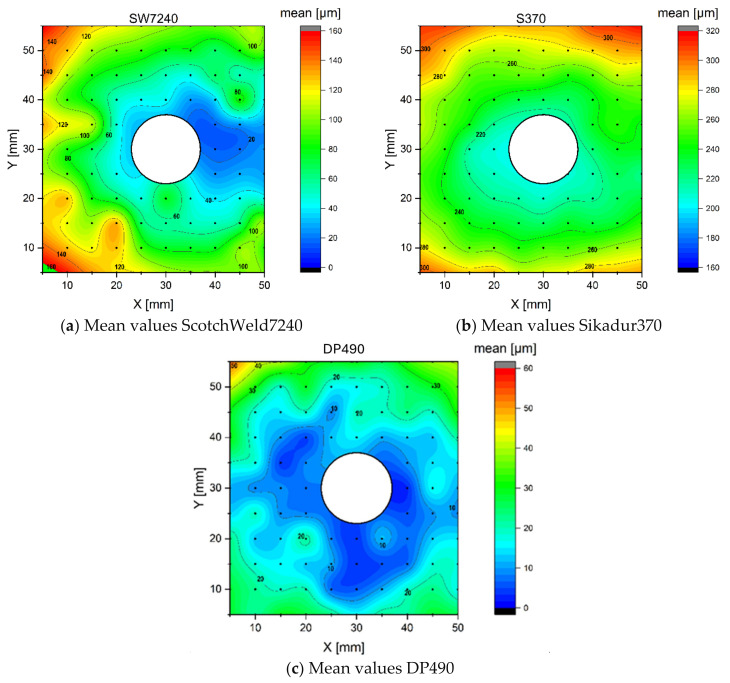
Results of the adhesive layer thickness measurements.

The results of the adhesive thickness measurements are shown in Figure 6. The mean values were obtained by averaging three measurements and the accuracy and repeatability were assessed using the standard deviation.

For the SW7240 epoxy (Figure 6a), the thickness ranged from approximately 20 µm near the center axis to 160 µm at the outer corners, with a mean value of 84.34 µm. The thickness distribution appeared to be symmetrical about the centerline of the steel plate, with the smallest thickness observed between the bolt holes. The standard deviation of the measurements was generally around 1 µm. In contrast, the Sikadur370 epoxy (Figure 6b) showed the maximum thickness values peaking at 320 µm on either side of the steel plate, with smaller values around the drill hole (approximately 200 µm, average: 256.1 µm). The thickness distribution was symmetrical but more radially distributed compared to SW7240. The standard deviation was slightly higher, averaging 2.55 µm. The measurements for the DP490 epoxy (Figure 6c) showed a much lower thickness (average: 18.7 µm), with a single peak of around 50 µm at one corner.

### 3.3. CFD of the Hybrid Joints

#### 3.3.1. S370

For the sand particle epoxy adhesive, a force of 62.62 kN was achieved at a final thickness of 200 μm with a downward substrate velocity of 1 mm/s. This result closely matches the experimental data, where an estimated force of 65 kN was recorded at the same thickness. The achievement of the estimated experimental force at the desired adhesive thickness can be attributed to the adhesive’s high viscosity, as indicated by its rheological characterization.

**Figure 7:**
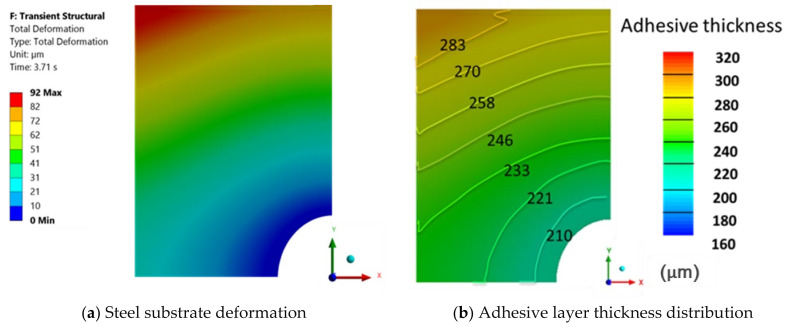
Numerical results obtained for S370 adhesive with a moving wall velocity of 1 mm/s.

The deformation of the metal substrate is depicted in Figure 7. By incorporating the final thickness achieved, the thickness distribution of the adhesive layer is obtained, as shown in Figure 7b. The simulation for this adhesive produced the most precise and reliable results, with convergence assured throughout the entire fluid compression process.

The thickness increases radially, being greater in the vertical axis, where the confined space is larger and, therefore, higher pressure is generated. In the end, the greatest thickness obtained was 292 μm and the average value was 246 μm, closely aligning with the experimental average value of 256 μm. The difference between the average adhesive thickness value was a mere 4.07%.

**Figure 8:**
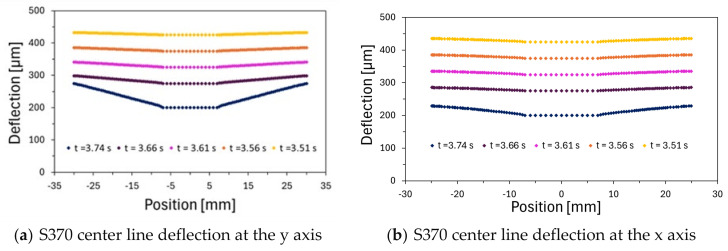
S370 deflection curves.

Figure 8 shows the deflection curves on both axes of the deformation plane, where it is possible to see the greater deflection on the vertical axis. Significant deflections appear at the very end of the compression process, for thicknesses thin enough to generate forces capable of deforming the material. In fact, it can be stated that, for this adhesive, until the force of 62.08 kN was reached, the gradient of deformation was unsubstantial.

The final deflection achieved, caused by the fluid’s rheological behavior, resembles the shape that would result from the application of a central force on the substrate, such as that exerted by a bolt. These results lead us to conclude that the fluid’s rheological behavior is the primary factor influencing the substrate’s deflection, thereby corroborating the assumed simplifications.

#### 3.3.2. DP490

The analysis of the particle-free adhesive focuses solely on the deformations of the metal substrate. This approach is necessary due to the chosen mesh size of 80 microns, as smaller mesh sizes resulted in memory storage issues. For this mesh size, accurate results are assured only up to a final thickness of 80 μm. Given that the finite volume method relies on the center point information of a fluid element, the results up to a thickness of 40 μm can be considered reasonable. Below this value, the results become unreliable. Therefore, it is not possible to accurately determine the distribution of the adhesive layer thickness for experimentally obtained values. Instead, the final deformation of the substrate at the smallest thickness ensuring numerical reliability (or upon reaching the estimated normal force of 65 kN) was analyzed. Given the achievement of very thin experimental thicknesses, the flow of this adhesive was analyzed at various compression speeds. This approach was necessary because the compression speed proportionally affects the normal force generated by the fluid.

**Figure 9:**
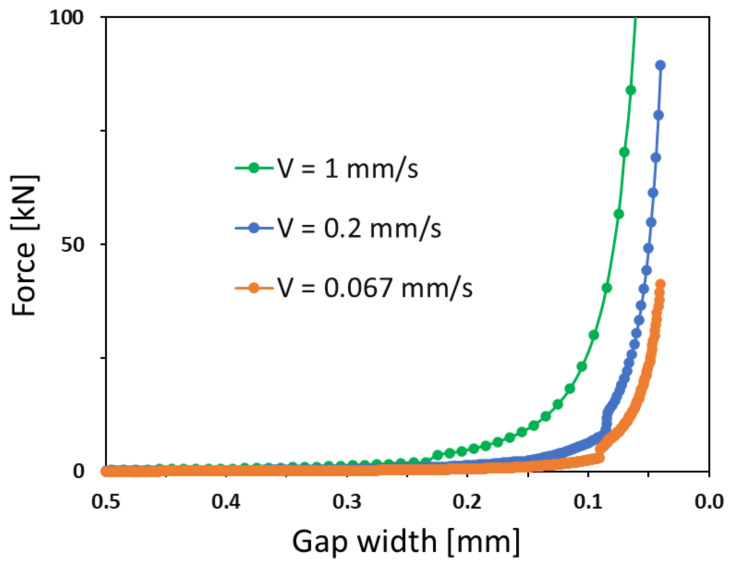
DP490 normal force produced for different velocities.

Figure 9 illustrates the variation in the normal force across the final range of gap widths for different compression speeds. It is evident that the lower the compression speed, the thinner the adhesive layer at which the estimated clamping force is achieved.

At a downward substrate velocity of 1 mm/s, a force of 70.37 kN was attained at the final adhesive thickness value of 70 μm. Additionally, the numerical deformation of the metal substrate remained constant up to a final thickness of 50 μm, despite a notable increase in the normal force observed within this range. This velocity yielded the closest correlation between the average substrate deformation and the adhesive layer thickness, with a recorded relative difference of 15.0% between these two values. Finally, it should be noted that from the final thickness of 40 μm, the deformation of the substrate increases sharply to a maximum of 337 μm.

**Figure 10:**
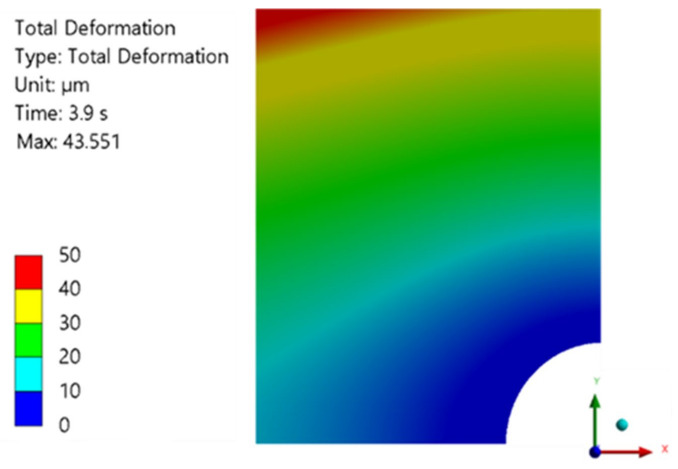
Numerical results obtained for DP490 adhesive with a moving wall velocity of 1 mm/s.

The deformation obtained is shown in Figure 10, which closely resembles the thickness gradient observed in the experimental adhesive layer, whose maximum value is close to 50 μm.

For a downward substrate velocity of 0.2 mm/s, a force of 65.33 kN was reached at a final thickness value of 50 μm. For this value, the deformation of the metal substrate reaches a maximum value of 71 μm, which is slightly above the deformation observed at a downward velocity of 1 mm/s for the same thickness.

**Figure 11:**
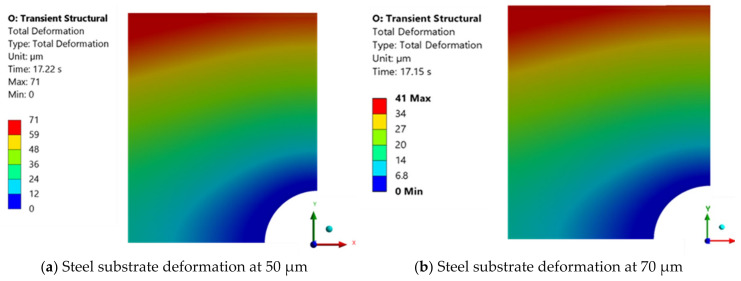
Numerical results obtained for DP490 adhesive with a moving wall velocity of 0.2 mm/s.

For comparison purposes, the deformation of the metallic substrate at the adhesive layer thickness of 70 μm was also obtained and is displayed on the right side of Figure 11. The result for this thickness value is virtually identical to the outcome obtained at a velocity of 1 mm/s for the same final thickness of the adhesive layer.

For thickness values below 50 μm, the deformation of the substrate increases significantly, reaching a maximum value of 130 μm at a thickness of 40 μm.

**Figure 12:**
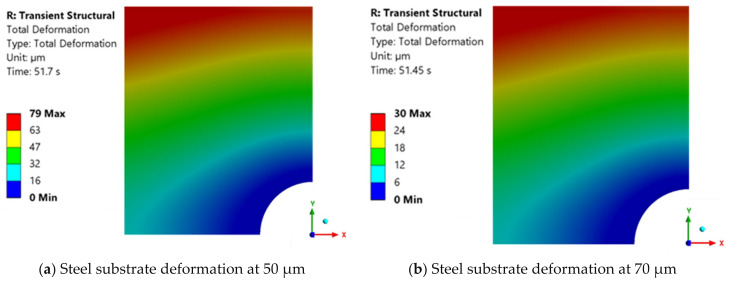
Numerical results obtained for DP490 adhesive with a moving wall velocity of 0.067 mm/s.

For a downward substrate velocity of 0.067 mm/s (or 4 mm/min), a force of 66.24 kN was reached at a final thickness value of 30 μm. For this final thickness value, the numerical results cannot be considered reliable, as the maximum deformation value obtained is 268 μm, which is far beyond the range of experimental values. Therefore, the deformations for the final thicknesses of 50 μm and 70 μm are presented below. For these values, maximum deformation values of 79 μm and 30 μm were obtained, as shown in Figure 12.

The result for the final adhesive layer thickness of 50 μm closely resembles the outcome obtained at a higher velocity of 0.2 mm/s. Similarly, for the final thickness of 70 μm, the results align with those observed at the other velocities for the same thickness value.

#### 3.3.3. SW7240

**Figure 13:**
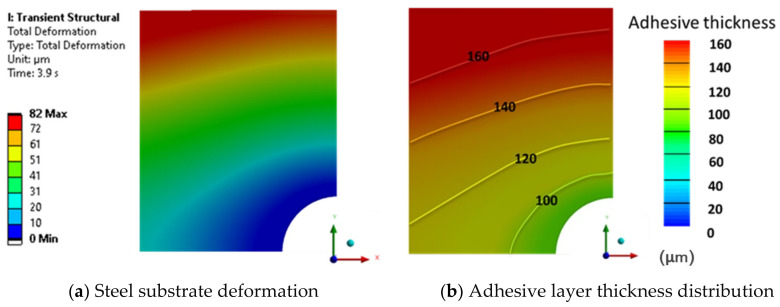
Numerical results obtained for SW7240 adhesive with a moving wall velocity of 1 mm/s.

For the glass particle adhesive, a force of 61.03 kN was reached at a final thickness value of 90 μm at a downward substrate velocity of 1 mm/s. The steel deformation gradient and the adhesive layer thickness distribution can be found in Figure 13. Although the maximum value of the adhesive layer thickness closely matches the experimental results, the minimum thickness value does not. Therefore, an effort was made to achieve a lower thickness value by reducing the substrate velocity.

For a downward substrate velocity of 0.2 mm/s, a force of 65.31 kN was reached at a final thickness value of 55 μm. The mean numerical value obtained for the thickness of the adhesive layer was 84.4 μm, resulting in a relative difference of 27.1% between the numerical and experimental results, which recorded a mean value of 61.5 μm. This discrepancy is attributed to the crushing of the particles; in the region to the left, where the particles remained intact, there is closer alignment between the results, as later discussed in Section 4.2.

The highest substrate deformation gradient was recorded for this adhesive, despite its low viscosity throughout the deformation rate, as seen in its rheological characterization. This may be due to its higher power index, n, which delays the adhesive’s less viscous behavior.

**Figure 14:**
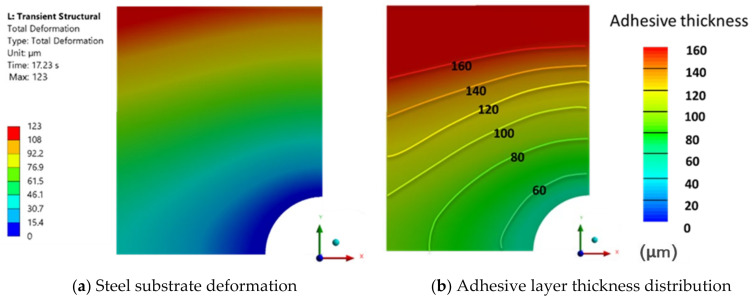
Numerical results obtained for SW7240 adhesive with a moving wall velocity of 0.2 mm/s.

**Figure 15:**
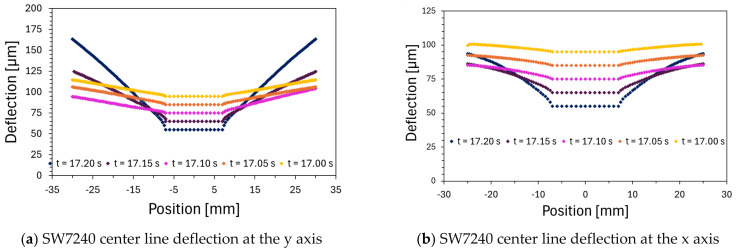
SW7240 deflection curves.

For this case, the gradient of the adhesive layer thickness increases significantly after passing the minimum thickness value of 75 μm, increasing even more when passing the thickness from 65 to 55 μm. Once again, the role of the adhesive’s rheology, responsible for the curvature felt in the substrate, is reinforced. Figure 14a,b, along with Figure 15a,b, respectively, illustrate the deformation of the metal substrate, the thickness distribution of the adhesive, the deflection of the center line along the vertical axis, and the deflection along the horizontal axis of the substrate.

## 4. Discussion

### 4.1. Experimentally Determined Adhesive Layer Thickness

Variations in the adhesive layer thickness were observed among the different adhesives. SW7240 exhibited a thickness ranging from 20 µm to 160 µm, with an average of 84 µm, while Sikadur370 had an average thickness of 256 µm, and DP490 averaged at 19 µm. Further measurements for the DP490 epoxy revealed an even lower thickness, with an average of 18.7 µm, and a single peak of around 50 µm at one corner. Additionally, for the acrylic DP8425, the thicknesses were notably smaller, averaging at 45.9 µm.

When considering the adhesives in terms of squeeze-out resistance, low-viscosity adhesives such as DP490 are often found to be prone to excessive squeeze-out, requiring precise control to prevent displacement. Medium-viscosity adhesives such as SW7240 are expected to provide a balance between flowability and resistance, making them suitable for applications where both good coverage and some resistance to squeeze-out are required. Conversely, high-viscosity adhesives such as Sikadur370 and DP8425 are expected to provide significant resistance to squeeze-out.

The adhesive film thickness measurements revealed the influence of the filler content and size on the bonding process for different adhesives. The filler content and size influenced the film thickness, with larger fillers resulting in thicker films. The viscosity played a role in the thickness, generally increasing with higher viscosity, although DP8425 deviated from this trend. SW7240, containing large glass beads, showed variable thicknesses, probably due to the bead spacing. Sikadur370 showed consistently thicker layers due to its high sand content, probably acting as a spacer. In contrast, DP490, with small filler particles, showed thinner, more uniform layers, allowing for closer bonding between the surfaces. Overall, the filler properties had a significant effect on the adhesive layer thickness, highlighting the importance of selecting adhesives with appropriate filler properties for specific applications.

### 4.2. Comparison between Experimental and CFD Results

**Figure 16:**
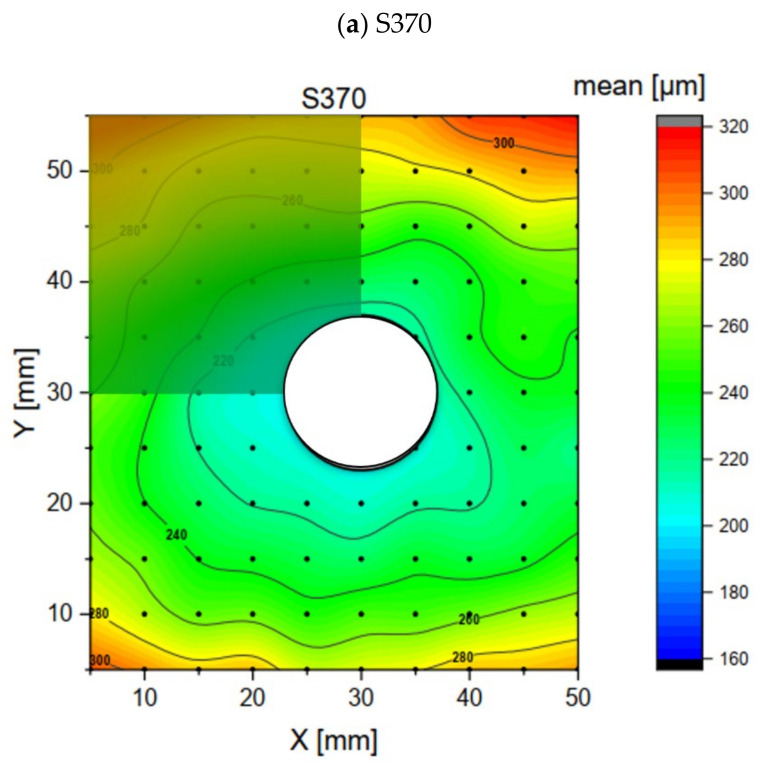

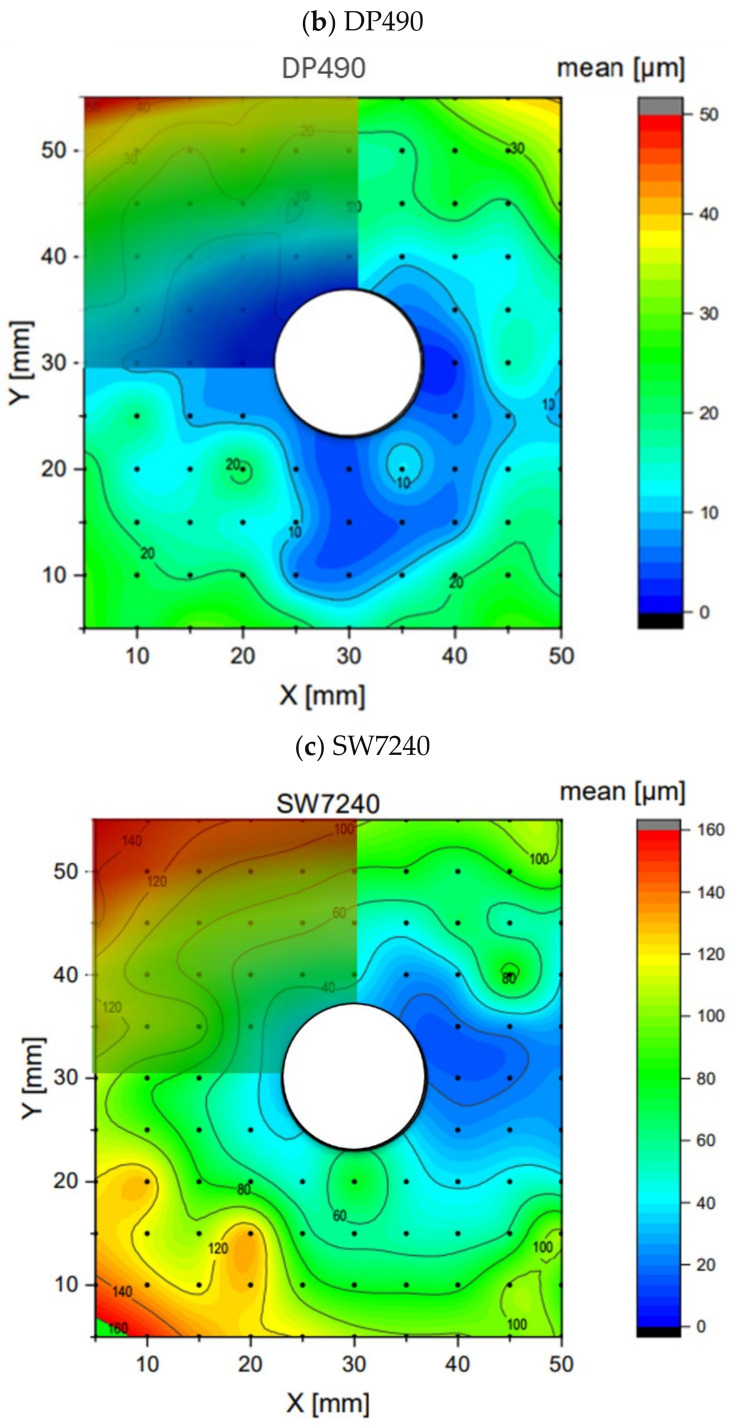
Distribution of adhesive thickness in hybrid joints: a comparison between the numerical and experimental results. The small square in the top left of each image shows the numerical results, while the larger square with the bolting hole presents the experimental data.

As shown in Figure 16, for the sand particle adhesive, the numerical results are practically identical to the experimental ones. The results are displayed in the first quadrant of the experimental data, as this quadrant best represents the simulation and the selected boundary conditions. The simulation was conducted for a geometry with a single bolt, whereas the experimental setup included five bolts within the same geometry.

The results suggest that using a velocity of 1 mm/s provides a good approximation of the reality. This hypothesis is plausible considering the two phases of adhesive compression: one where minimal mechanical interference occurs from the adhesive particles and another starting at a 200 μm thickness, involving ineffective efforts to overcome particle resistance. Additionally, there are other ineffective periods when the clamping tool is loosened. These findings reinforce the validity of the numerical model developed.

For the particle-free adhesive, the result that best portrays the experimental scenario is associated with a substrate descending velocity of 1 mm/s. At this velocity, the substrate achieves the deformation value for the smallest adhesive layer thickness, with the maximum deformation of 44 μm, closely matching the maximum experimental adhesive thickness gradient value of nearly 50 μm. Interestingly, for this thickness, the deformations at the lower velocities of 0.2 and 0.067 mm/s were greater than those at 1 mm/s.

The results point to the occurrence of the adhesive phase migration phenomenon, hypothesized from the experimental data interpretation. The exponential increase in substrate deformation for thickness values below 50 μm, observed at both 1 mm/s and 0.2 mm/s for a thickness value of 40 μm, suggests the redistribution and displacement of the adhesive from this point onward. This phenomenon causes the adhesive to flow to areas of lower pressure, such as the bolt hole and the free edge, leaving areas with little or no adhesive, as observed experimentally around the bolt area. This rearrangement of the adhesive layer affects the pressure field acting on the substrate interface, preventing the normal progression of deformation that would occur if the adhesive layer remained intact along the joint.

In summary, the results for thicknesses beyond 50 μm, at the 40 μm mark, illustrate the substrate’s deformation in the absence of the migration phenomenon. Since such deformations are not observed experimentally, they can be attributed to the adhesive phase migration phenomenon. The close correspondence between the numerical deformation and the experimental results implies that the numerical deformation recorded represents the last deformation suffered by the substrate. This strongly suggests that the migration of the adhesive occurred during the last stages of compression.

For the adhesive with glass particles, a downward substrate speed of 0.2 mm/s produced the best results. The deformation gradient obtained was within the range of the adhesive layer thickness values, varying up to a maximum value of 123 μm. In turn, good similarity to the experimental results was obtained with regard to the distribution of the thickness of the adhesive layer itself.

This adhesive underwent the particle crushing process, a phenomenon that is impossible to recreate numerically, as the crushed particles alter the adhesive’s rheology. Despite this challenge, the results obtained are positive and closely match the experimental data. Two possible reasons could explain this outcome.

The first is the large heterogeneity in the particle size observed microscopically in the experimental work. This variation in particle size accounts for the selective crushing of larger particles while smaller ones remain intact. The numerical results suggest that only a small proportion of the particles were crushed, minimally altering the adhesive’s rheology, thus explaining the agreement between the simulated and experimental results. Second, the radial migration of particles within the joint could also be a factor. The findings indicate that the majority of the glass particles likely migrated to areas of lower pressure, specifically at the ends of the joint. This migration is particularly notable on the left side, which represents the beginning of the joint and thus exhibits lower pressure compared to the right side, where the joint continues. The right side, with its more confined space, presents higher-pressure conditions that inhibit such migration. The boundary conditions used in the simulation accurately represent this scenario, where all sides are exposed to the outside environment. This suggests that, under these conditions, the adhesive’s rheology plays the primary role in the distribution of the adhesive layer. The glass particles, when moving to these areas, exhibit minimal mechanical resistance, allowing them to remain intact and play a lesser role than the adhesive rheology. This migration is facilitated by the compressive strength and sphericity of the glass particles, which enable them to move within the joint.

## 5. Conclusions

This study reveals significant variability in the adhesive layer thickness among different adhesives, ranging from an average of 18.7 µm for DP490 to 256 µm for Sikadur370. This variability is largely influenced by the viscosity of the adhesive, which plays a crucial role in determining both the layer thickness and squeeze-out resistance. Low-viscosity adhesives like DP490 are prone to excessive squeeze-out and require precise control, while medium-viscosity adhesives such as SW7240 offer a balance between flowability and squeeze-out resistance. High-viscosity adhesives like Sikadur370 provide significant resistance to squeeze-out.

The filler content and size have been identified as key factors influencing the final adhesive film thickness. Larger fillers generally result in thicker adhesive films, while high filler content, such as the sand in Sikadur370, acts as a spacer, leading to consistently thicker layers. Conversely, small filler particles, as found in DP490, allow for thinner, more uniform layers and closer bonding between surfaces.

The numerical model developed for the CFD simulation demonstrates capabilities in predicting the adhesive layer thickness in bolted hybrid joints with reasonable accuracy, particularly for adhesives containing sand particles. The simplifications incorporated into the CFD model, such as treating the substrate as a rigid body and using the velocity instead of the force for bolt tightening simulation, proved effective in producing meaningful results.

The investigation highlights that decreasing the downward velocities of the substrates resulted in greater deformations, despite the less pronounced evolution of the normal force produced by the adhesive.

Furthermore, the research emphasizes the importance of accurately defining the boundary conditions and joint geometry. When considering a hybrid joint with a single bolt, allowing the adhesive to exit at all ends of the joint, the results were generally more similar to the areas associated with the upper and lower left quadrants of the experimental results, where the adhesive tended to flow.

Based on the conclusions of this study, it is recommended to ensure improved fixing points at the ends of the joint, where the adhesive layer thickness tends to be greater, to achieve the more uniform distribution of the adhesive layer thickness. Additionally, careful control of the torque applied to tighten the bolts is advised, as different tightening times can result in more pronounced thickness variations along the joint. By applying the torque more gradually, the more even distribution of the adhesive layer can be achieved.

This study underscores the critical role of adhesives’ rheological behavior in determining the thickness of the adhesive layer in hybrid bolted joints. It is noteworthy that the achieved thicknesses in hybrid bolted joints are generally lower than those in conventional adhesive joints, emphasizing the unique characteristics of this joining method.

Looking forward, several areas for future research emerge from this study. Further investigation aimed at improving the simulation accuracy for very thin adhesive layers is warranted. Consideration of the viscoelasticity of the adhesive may be relevant for the final stages, where the length scale may enhance the elastic response of the fluid [65]. This is possible in software such as OpenFoam-v2312 and is expected to be possible in the upcoming versions of the Ansys Fluent 2025 R1 software used in this study. Additional studies on the effects of particle crushing and its impact on the adhesive rheology during the bonding process could enhance our understanding and the model’s accuracy. Moreover, the exploration of methods to overcome the convergence issues in CFD simulations for thin adhesive layers could improve the tool’s applicability across a wider range of scenarios.

In conclusion, this research highlights the complex interplay between the adhesive properties, joint design, and simulation techniques in predicting and optimizing the adhesive layer thickness in hybrid bolted joints. The findings contribute significantly to the understanding of the adhesive behavior in these joints and provide a foundation for the further refinement of predictive models and joint design optimization.

## Figures and Tables

**Table 1:** Adhesive properties (experimentally determined, except those marked *, which were obtained from the corresponding technical datasheet) [34].

Property	Unit	SW7240	S370	DP490
**Lap shear strength**				
On blasted surface	[N/mm^2^]	29.3 ± 1.5	25.1 ± 0.6	31.7 ± 1.2
On mill scale	[N/mm^2^]	29.6 ± 0.8	29.3 ± 0.9	16.8 ± 1.4
**T_g_**	[°C]	75	73	69
**Bulk properties**				
E modulus	[N/mm^2^]	1832 ± 54	3582 ± 347	1954 ± 46
Poisson’s ratio		0.40 ± 0.02	0.31 ± 0.09	0.38 ± 0.05
Maximum strength	[N/mm^2^]	25.9 ± 1.0	25.3 ± 0.7	36.4 ± 0.6
Elongation at break	[%]	1.9 ± 0.3	1.2 ± 0.3	3.3 ± 1.2
**Inorganic fillers**				
Material (majority)		Glass beads	Sand	Unknown
Mass percent	[%]	28.6	62.8	1.5
Diameter	[µm]	176 ± 10 (160–300) *	197 ± 40	<10
**Load capacity**	[kN]	351.5 ± 9.5	394.2 ± 5.4	329.4 ± 19.1

**Table 2:** Boundary conditions.

Boundary Name	Boundary Type
Moving substrate	UDF
Fixed substrate	Wall
Outlet	Pressure outlet
Symmetry face (3D)	Symmetry
Axis edge (2D)	Axisymmetry

**Table 3:** Scheme of results to be analyzed.

Adhesive	Substrate Velocity	Targeted Final Gap Width
S370	V = 1 mm/s	200 μm
DP490	V = 1 mm/s	10 μm
	V = 0.2 mm/s	
	V = 0.067 mm/s = 4 mm/min	
SW7240	V = 1 mm/s	40 μm
	V = 0.2 mm/s	

**Table 4:** CFD solution methods.

Item	Numerical Method
Viscous model	Laminar
Pressure–velocity coupling	COUPLED
Spatial discretization of gradient	Least squares cell-based
Spatial discretization of pressure	Second-order
Spatial discretization of momentum	Second-order upwind
Transient formulation	Second-order implicit

**Table 5:** Fitted parameters in the rheological constitutive model for the adhesives.

Property	Unit	SW7240	S370	DP490
$\eta_{0}$	[Pa·s]	3466.5	43,036.5	17,348.0
$\eta_{\infty }$	[Pa·s]	77.0	495.2	31.1
λ	[s]	101.12	85.27	104.30
n	[–]	0.373	0.168	0.226

**Figure A1:**
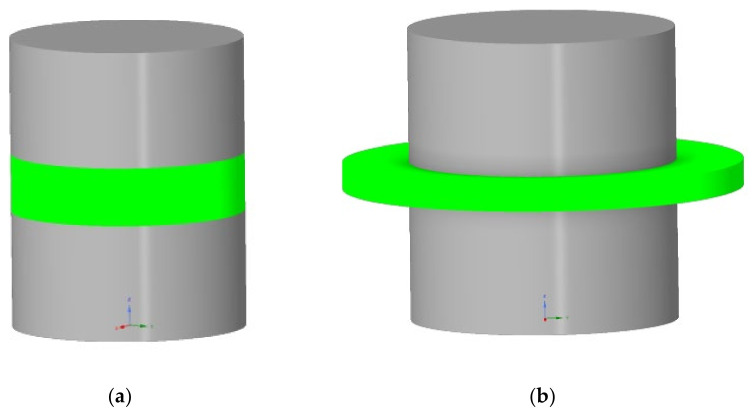
Adhesive squeeze flow before (**a**) and after (**b**) the squeezing process.

**Figure A2:**
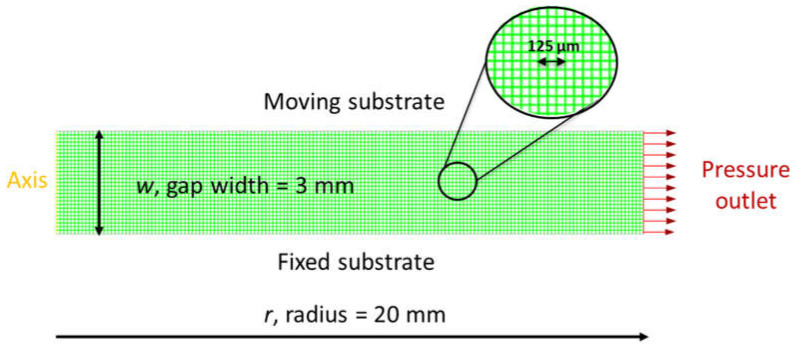
Squeeze flow axisymmetric model.

**Figure A3:**
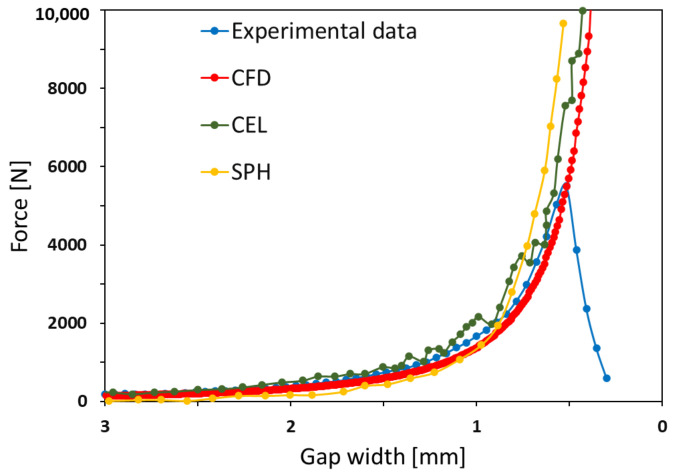
Validation of the numerical model by a comparison with numerical and experimental results from the literature [50].

**Figure A4:**
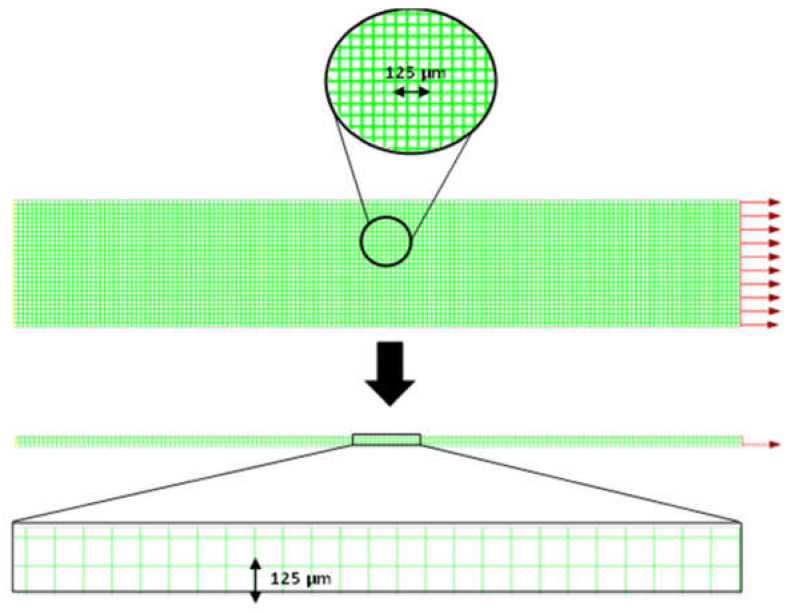
Squeeze flow axisymmetric model mesh before (**top**) and after (**bottom**) its compression.

## Data Availability

The original contributions presented in the study are included in the article; further inquiries can be directed to the corresponding authors.

## Appendix A. Substrate Movement

The UDF controlling the downward movement of the adhesive–substrate interface initiates with a start-up period where the velocity increases linearly with time until reaching a specified value. It then remains constant until the final adhesive thickness is achieved, after which the velocity passes to zero. The UDF works as follows:

#include "udf.h"

#include "dynamesh_tools.h"

DEFINE_CG_MOTION(wallmov,dt,vel,omega,time,dtime)

{

Thread *t;

face_t f;

NV_S (vel, = , 0.0);

real max_velocity = -0.001;

real acceleration_time = 1.0;

real final_thickness_time = 3.8;

if (time < acceleration_time)

{

vel[2] = (max_velocity / acceleration_time) * time;

}

else if (time >= acceleration_time && time < final_thickness_time)

{

vel[2] = max_velocity;

}

else if (time > final_thickness_time)

{

vel [2] = 0;

}

Message("Current velocity: %f\n", vel [2]);

Message("Current z coordinate: %f\n", DT_CG (dt) [2]);

}

## Appendix B. Validation Process

### Appendix B.1. Squeeze Flow Model

The model aims to validate the chosen methods by comparing the normal force generated by the pressure exerted by the fluid on the substrate during compression in its squeeze flow with those documented in the literature.

The model selected to represent the fluid domain during the squeeze flow process is configured in the software as an axisymmetric problem. This configuration corresponds to an initial geometry representing a slice of the cylindrical shape that the fluid assumes prior to flowing, as can be seen in Figure A1.

The geometry considered for this model is rectangular, with its height decreasing over time due to the movable boundary condition applied to the top horizontal edge, representing the adhesive–substrate interface. The left vertical edge represents the axis of revolution, while the right vertical edge marks the boundary delimiting the end of the confined space between the substrates and is thus assigned an outlet condition, as indicated by the adjacent red arrows. The lower horizontal edge, with an assigned wall condition, represents the fluid in contact with the fixed metal substrate.

The numerical model is illustrated in Figure A2, identified by the measure r, which denotes the radius of the geometry. The normal force exerted by the fluid when compressed, F, will be calculated using a UDF that integrates the pressure along the radial direction, considering the chosen mesh size.

### Appendix B.2. CFD Model Validation

#### Appendix B.2.1. Normal Force Comparison with Literature Results

The aforementioned numerical model overlooks the substrate wall roughness, assumes the fluid to be inelastic, and considers perfect geometric adhesive disposition. Thus, with the aim to establish the credibility of the developed numerical models, the two-dimensional axisymmetric squeeze flow case was tested and compared with the literature results.

The results obtained are consistent with the experimental results reported by Huf et al. [50]. An initial adhesive thickness of 3.0 mm was used, a downward substrate velocity of 1.0 mm/s and a geometry radius of 20 mm were selected, and a final adhesive thickness of 0.3 mm was attained. In this study, the adhesive employed was a one-part epoxy labeled Sika Power 498. This adhesive’s rheological characterization was fitted by a non-Newtonian power-law fluid whose viscous behavior followed Equation (3):

(A1) $$\eta =k\cdot \dot{\gamma^{n-1}},$$

where η represents the viscosity; k is the flow consistency index, such that k = 9000 Pa; and n is the the flow behavior index, such that n=0.55.

The graph comparing the numerical results obtained with the available experimental results is displayed in Figure 9. As illustrated, the results validate the numerical results. The slight discrepancies observed between the numerical and experimental results are most likely attributable to the elasticity of the fluid, which is not accounted for in this study.

Figure A3 also presents a comparison of the numerical results obtained in this study with the numerical results from Huf et al. [50] obtained through the CEL and SPH methods. This comparison highlights the efficacy of the selected numerical approaches, underscoring the robustness and reliability of the methods used in this study.

#### Appendix B.2.2. Mesh Analysis

The mesh of the fluid domain is crucial for the accuracy of the obtained solutions, given that the problem begins with very thin adhesive layers and finishes with even thinner layers. For this reason, the normal force values produced with the two-dimensional squeeze flow model of the Sika Power 498 adhesive were compared across consecutively refined mesh sizes. This comparison aimed to determine the mesh size at which the results stabilize, indicating that they are independent of the mesh size. The mesh used is a square; hence, its size is also referred to as its height.

The results are presented in Table A1, where the deviation for each mesh size is calculated by subtracting the force value of the previous mesh size from the force value of the current mesh size and then dividing it by the force value of the current mesh size:

(A2) $$\frac{\left|Force_{\;Coarse}-Force_{\;Fine}\right|}{Force_{\;Fine}}\times 100$$

As can be seen, the 80 μm mesh size yields results that are very similar to those for the 100 μm mesh, with a maximum difference of just 1.62%. Within this tolerance range, it can be concluded that the results for this mesh size are independent of the mesh size. However, it is important to note that as the thickness of the adhesive layer decreases and the forces increase with each iteration, the deviation between different mesh sizes also increases. This emphasizes the need for the careful consideration of mesh size selection to balance the computational efficiency and accuracy, especially in scenarios where significant changes in force are anticipated.

**Table A1:** Normal force results for different mesh sizes at different gap width values.

Cell Size [μm]	125		100		80
Gap Width [mm]	Force [kN]	Deviation [%]	Force [kN]	Deviation [%]	Force [kN]
2.00	0.33	0.23	0.33	0.18	0.33
1.00	1.39	0.45	1.39	0.03	1.39
0.50	5.94	0.035	5.94	1.09	6.00
0.30	19.71	5.75	20.91	1.62	21.26

For a mesh size of 80 μm, the numerical results were compared with the experimental ones in Table A2; as such,

(A3) $$\frac{\left|Force_{\;Experimental}-Force_{\;Numerical}\right|}{Force_{\;Experimental}}\times 100$$

**Table A2:** Experimental and numerical normal force comparison.

Gap Width [mm]	Experimental Force [kN]	Numerical Force [kN]	Deviation [%]
2.5	0.22	0.21	4.47
2.0	0.34	0.33	3.05
1.5	0.62	0.60	3.81
1.0	1.43	1.39	2.46
0.5	5.88	6.00	2.02

Another consideration when selecting the mesh size is the computational time. The two-dimensional model is advantageous in estimating the computational cost relative to the mesh size, as the three-dimensional model requires significantly more computational resources. As evident from the results presented below (Table A3), a mesh size of 40 μm would be ideal to conduct the simulations, as, beyond this value, the computational time increases significantly. However, it was observed that for the three-dimensional model, mesh sizes smaller than 60 microns resulted in memory storage issues. Therefore, a compromise was made, and a mesh size of 80 μm was chosen to balance the computational efficiency with the memory constraints.

**Table A3:** Computational time for different mesh sizes.

Cell Size [μm]	Computational Time [min]
120	24
100	25
80	28
60	30
40	37
30	120
20	180

For this mesh, precise results can be guaranteed up to a final adhesive thickness of 80 μm. Based on the principles of the finite volume method, which relies on the information at the center point of the element, it can be considered that the information up to this point is reasonably accurate. For a mesh size of 80 μm, this point corresponds to a final adhesive thickness of 40 μm. Below this thickness value, the results cannot be considered reliable and, consequently, will not be analyzed.

For the mechanical model, the results for different mesh sizes are practically identical up to a global mesh size of 1 mm. Additionally, significant computational time savings are achieved by adjusting the height of the elements, which do not need to be as refined as those used in the CFD model.
